# Novel para-aortic cardiac assistance using a pre-stretched dielectric elastomer actuator

**DOI:** 10.1093/icvts/ivae027

**Published:** 2024-02-28

**Authors:** Silje Ekroll Jahren, Thomas Martinez, Armando Walter, Francesco Clavica, Paul Philipp Heinisch, Eric Buffle, Markus Martin Luedi, Jürgen Hörer, Dominik Obrist, Thierry Carrel, Yoan Civet, Yves Perriard

**Affiliations:** Integrated Actuators Laboratory (LAI), École polytechnique fédérale de Lausanne (EPFL), Neuchâtel, Switzerland; ARTORG Center for Biomedical Engineering Research, University of Bern, Bern, Switzerland; Integrated Actuators Laboratory (LAI), École polytechnique fédérale de Lausanne (EPFL), Neuchâtel, Switzerland; Integrated Actuators Laboratory (LAI), École polytechnique fédérale de Lausanne (EPFL), Neuchâtel, Switzerland; Integrated Actuators Laboratory (LAI), École polytechnique fédérale de Lausanne (EPFL), Neuchâtel, Switzerland; ARTORG Center for Biomedical Engineering Research, University of Bern, Bern, Switzerland; Department of Congenital and Pediatric Heart Surgery, German Heart Center Munich, Technical University of Munich, Munich, Germany; Division of Congenital and Pediatric Heart Surgery, University Hospital of Munich, Ludwig-Maximilians-University, Munich, Germany; Department of Cardiology, Bern University Hospital Inselspital, University of Bern, Bern, Switzerland; Department of Anaesthesiology, Bern University Hospital Inselspital, University of Bern, Bern, Switzerland; Department of Congenital and Pediatric Heart Surgery, German Heart Center Munich, Technical University of Munich, Munich, Germany; Division of Congenital and Pediatric Heart Surgery, University Hospital of Munich, Ludwig-Maximilians-University, Munich, Germany; ARTORG Center for Biomedical Engineering Research, University of Bern, Bern, Switzerland; Department of Cardiac Surgery, University of Zurich, Zurich, Switzerland; Integrated Actuators Laboratory (LAI), École polytechnique fédérale de Lausanne (EPFL), Neuchâtel, Switzerland; Integrated Actuators Laboratory (LAI), École polytechnique fédérale de Lausanne (EPFL), Neuchâtel, Switzerland

**Keywords:** Dielectric elastomer actuator, Cardiac assist device, Counterpulsation, *in vivo* experiment

## Abstract

**OBJECTIVES:**

We propose an evolution of a dielectric elastomer actuator-based cardiac assist device that acts as a counterpulsation system. We introduce a new pre-stretched actuator and implant the device in a graft bypass between the ascending and descending aorta to redirect all blood through the device (ascending aorta clamped). The objective was to evaluate the influence of these changes on the assistance provided to the heart.

**METHODS:**

The novel para-aortic device and the new implantation technique were tested *in vivo* in 5 pigs. We monitored the pressure and flow in the aorta as well as the pressure–volume characteristics of the left ventricle. Different activation timings were tested to identify the optimal device actuation.

**RESULTS:**

The proposed device helps reducing the end-diastolic pressure in the aorta by up to 13 ± 4.0% as well as the peak systolic pressure by up to 16 ± 3.6%. The early diastolic pressure was also increased up to 10 ± 3.5%. With different activation, we also showed that the device could increase or decrease the stroke volume.

**CONCLUSIONS:**

The new setup and the novel para-aortic device presented here helped improve cardiac assistance compared to previous studies. Moreover, we revealed a new way to assist the heart by actuating the device at different starting time to modify the left ventricular stroke volume and stroke work.

**Figure:**
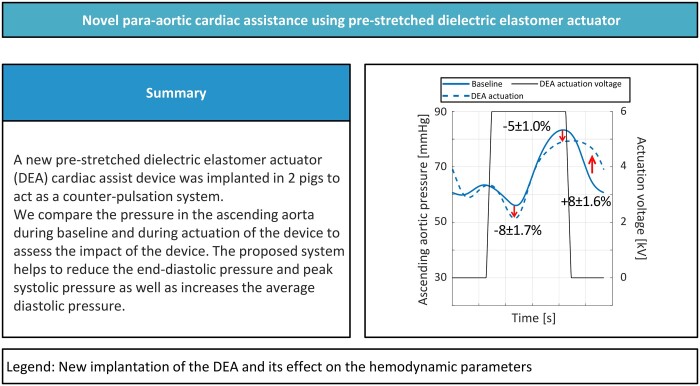

## INTRODUCTION

Heart failure (HF) is a condition characterized by a reduced ability of the heart to pump blood. Sixty-four million people were estimated to suffer from HF worldwide in 2017 and, due to its high prevalence, HF is considered a global pandemic. The costs projections associated with HF, for the year 2030, suggest that ∼70 billion dollars will be spent for HF in USA alone. For severe HF, heart transplant is considered the gold standard. Cardiac assist devices have been introduced as bridge to transplantation because of the shortage of heart donors. More recently, the interest of these devices has shifted towards a destination strategy [1, 2]. Nevertheless, cardiac assist devices as destination therapies are still unmet clinical needs [3].

Currently, Ventricular Assist Devices (VADs) with rotary pumps are the most common cardiac assist solutions [4]. They generate a constant flow and are characterized by high durability. However, due to the high shear stress generated by the rotating components on the blood, current VADs can cause haemolysis and thrombosis, which force patients to follow anticoagulation treatment for the whole duration of the cardiac support [5]. On the contrary, assist devices based on aortic counterpulsation (ACP) do not require anticoagulation treatment [6] and can help preserving (or even augment) the aortic pulsatile flow [7]. There exist several types of ACP devices that are named based on their location within the aorta. The ACP working principle is similar in all devices as they are designed to reduce the afterload during systole, and increase the coronary flow during diastole [8]. The ACP devices are still short-term solutions to bridge other options (e.g. VADs or patient recovery) in high-risk patients. This is mainly because of the high risk of ischaemia [9] associated with the ‘large’ ACP transcutaneous pneumatic drivelines.

Dielectric elastomer actuators (DEAs) emerge as a compelling alternative to existing assist devices, distinguished by their softness compared to rigid VADs and their sole reliance on electrical activation, compared to existing pneumatic ACP devices. These advantages could enable an implantable device and long-term cardiac support. DEAs consist of a hyperelastic dielectric membrane sandwiched between 2 compliant electrodes. Applying high voltage between the electrodes generates a Maxwell pressure that compresses the dielectric elastomers in the thickness dimension, enabling expansion in the other directions [10]. In our previous work [11], we already demonstrated the potential of our approach by showing the effects of the DEA as a cardiac assist device in porcine animal models. At the beginning of systole, the DEA is activated and can store more blood, thus decreasing the pressure in the ascending aorta. This decrease in pressure continues while the device is active. When the aortic valve closes, the DEA is deactivated, and the stored blood is released leading to an increase of pressure in the aorta. The effect is similar to the effect of intra-aortic balloon pumps (IABPs).

In [11], the DEA was implanted in the descending aorta, far from the aortic valve. Consequently, the pressure waves generated by the DEA underwent significant damping and energy losses before reaching the aortic valve [12]. Moreover, only a fraction of the total blood flow passed through the DEA (due to the supra-aortic vessels upstream of the DEA). The main reason that prevented DEA implantation in ascending aorta was the limited space, due to the shorter porcine ascending aorta (∼4 cm vs 8 cm of human aorta) [13]. The goal of the current study is to demonstrate that a pre-stretched soft DEA can support the heart by lowering the end-diastolic pressure and increasing early aortic diastolic pressure compared to previous versions [11]. Pre-stretching the DEAs is an easy and inexpensive technique that allows to increase the maximum electric field the DEA can sustain and limit its breakdown [14, 15]. This pre-stretch, results into a more stable and more performant DEA. In addition, by measuring the pressure–volume (PV) characteristics of the left ventricle, we want to show the effect of our assist device on the stroke volume and stroke work of the heart. Finally, we aim to demonstrate the pertinence of the new implantation technique to reproduce conditions closer to the human ones for the cardiac assist device.

## MATERIALS AND METHODS

### Ethical statement

This experiment presented in this paper was approved by the Commission of Animal Experimentation of the Canton of Bern, Switzerland (approval number BE14/2021).

### A pre-stretched dielectric elastomer actuator for cardiac assist device

**Figure 1:**
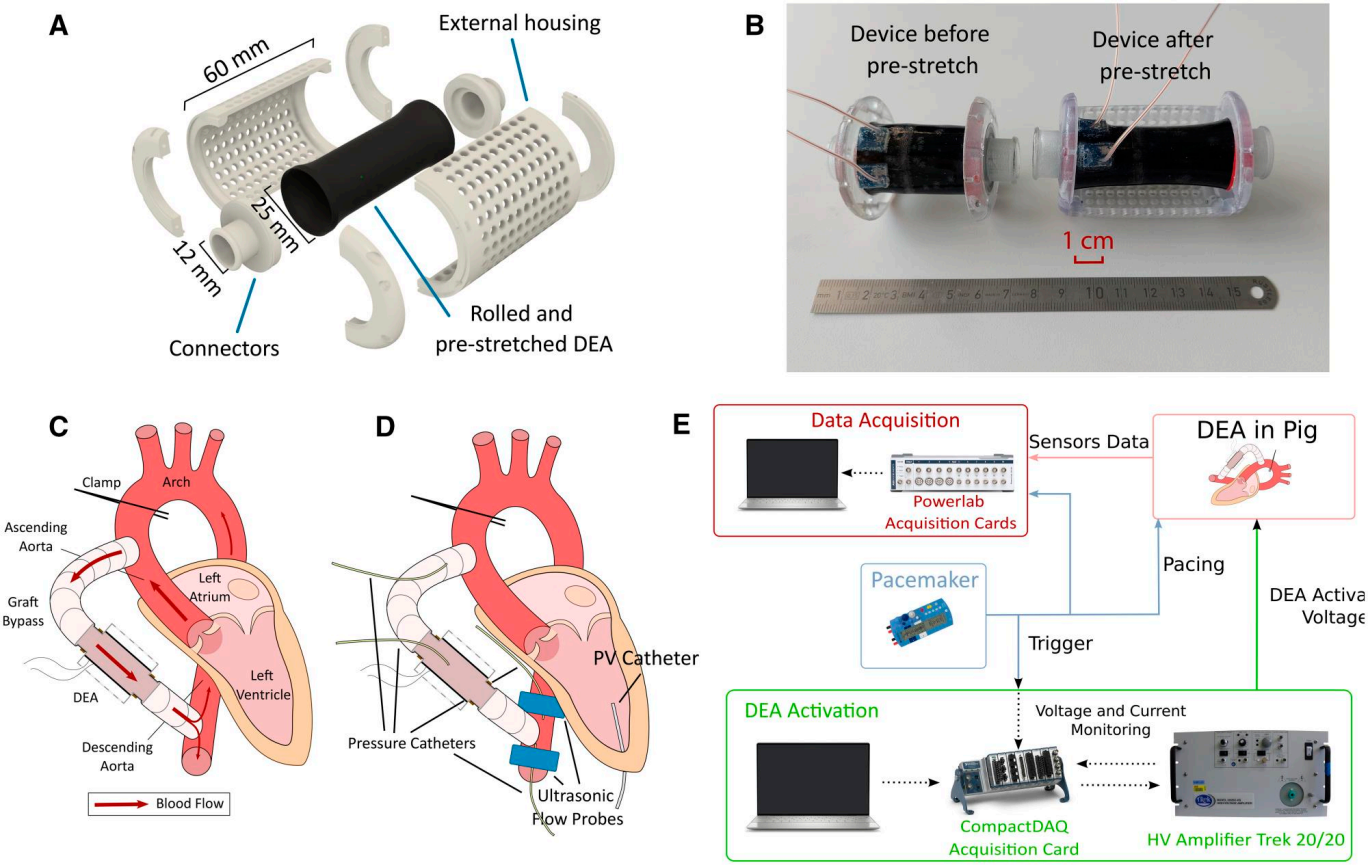
DEA and sensors implantation and hardware actuation and acquisition scheme. (**A**) Schematic of the DEA design. The connectors are there to enable anastomosis with the aorta and provide inner support for the DEA. The external housing helps protect the DEA from the outside environment and allows to apply the pre-stretch. (**B**) Picture of the device before and after pre-stretch is applied. The total length goes from 40 to 60 mm. (**C**) Schematic of the left section of the heart showing the different sections of the aorta as well as the graft bypass with the DEA. (**D**) Location of the different sensors used during the *in vivo* experiment. (**E**) Overview of the hardware setup used during the *in vivo* experiments. The DEA is activated at high voltages (kV) in synchronization with the pacing signal from the pacemaker through the CompactDAQ and the LabVIEW software. DEA: dielectric elastomer actuator; HV: high voltage; PV: pressure–volume.

The DEA cardiac assist device is based on a tubular DEA. The initial tube has a total length of 40 mm, a diameter of 25 mm and an overall thickness of ∼500 µm and is made of Elastosil 2030 (Wacker Chemie AG, Munich, Germany), a silicone elastomer. Compared to previous works, in this study, the DEA is pre-stretched axially and maintained in this position with an external housing. We were thus able to increase its length up to 60 mm, i.e. a 1.5 times pre-stretch. The full housing of the DEA, as shown in Fig. 1A and B, allows also to have a different diameter between the aorta and the DEA. By increasing the diameter of the DEA, the activation and deactivation of the device generate a higher displacement of blood volume and increases the effect on the cardiovascular system.

The PV characteristics of the DEA characterize the behaviour of the actuator and allow to estimate the volume displacement and the energy provided by the DEA during *in vivo* experiments. They were obtained through *in vitro* tests at different actuation voltages. The experimental testbench consists of a pneumatic system composed of a piston and a motor connected to the device and coupled to a pressure sensor (Baumer PBMN-25B12, Frauenfeld, Switzerland) [11, 16]. At different actuation voltages, the motor is moved to increase the pressure in the device, while the displacement is measured with a two-dimensional laser sensor (Gocator 2030, LMI Technologies, Vancouver, Canada) yielding the PV characteristics at constant voltage.

### Implantation of the device and measurements

An acute porcine model was used to test our device *in vivo* in *n* = 5 Edelschwein pigs ranging from 50 to 70 kg. The complete anaesthesia process for the pigs is described in the Supplementary Materials. For the *in vivo* experiments, a new surgery protocol has been developed. The thoracic cavity was accessed with an extended left sided thoracotomy (Hemi-clam-shell incision) and the pericardium was opened after administration of intravenous heparin. To implant the DEA, the aorta was partially clamped by a Satinsky clamp, 1st in the ascending aorta (close to the 1st branch of the aortic arch) and then in the descending aorta at the level of the diaphragm. Dacron Grafts (12–18 mm Gelweave, Vascutek Ltd, Inchinnan, UK) were sutured as end-to-side anastomosis to the ascending and the descending aorta and cut in half (Fig. 1C). The DEA was embedded in between the 2 remaining Dacron Grafts to allow exchange of the devices. After DEA implantation, the Satinsky clamps were removed. During the experimental testing of the DEA device, an aortic cross-clamp was placed just below the truncus brachiocephalicus to allow blood flow to be directed exclusively through the DEA device into the descending aorta. In case of exchange of the DEA device, the aortic cross-clamp was temporally removed. With this new configuration, the whole blood exiting the left ventricle passed through the graft and the DEA, followed by the descending aorta where the flow splits into 2 streams. One stream goes through the normal path going down the descending aorta and the other goes back up the descending thoracic aorta to supply the aortic arch and the aortic branches.

All the sensors are shown in Fig. 1D. Blood flow was measured with ultrasonic flow probes (Confidence, Transonic Systems, Inc., Ithaca, NY, USA) on each side of the connection between the graft and descending aorta to measure the total blood flow. Two water-filled pressure catheters (Xtrans, CODAN Pvb Critical Care GmbH, Forstinning, Germany) were inserted near these flow probes. Two additional pressure sensors were positioned in the DEA and in the ascending aorta as close as possible to the aortic valve. Finally, a PV catheter (Millar, ADInstruments, Houston, USA) was inserted in the left ventricle through the apex. The heart rate was controlled with a pacemaker. The pacemaker also acted as a trigger to synchronize the activation of the device with the heart.

All data were recorded through 2 acquisition consoles (Powerlab, ADInstruments, Houston, USA) and the DEA was actuating by a high voltage amplifier (Trek 20/20C, Advanced Energy, Denver, USA) controlled by a compactDAQ (National Instruments, Austin, USA) acquisition card as shown in Fig. 1E.

### Testing protocol

The testing protocol was identical to the one presented for the previous animal experiment [11].

**Figure 2:**
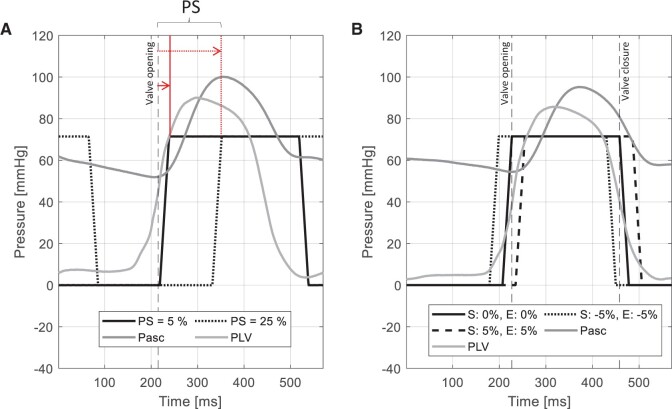
Description of the 2 different protocols for DEA actuation timing (amplitude scaled to pressure) compared to the left ventricular (PLV) and aortic pressures (Pasc): (**A**) phase shifting (PS, in % of heart cycle) where the start of the DEA actuation signal is shifted throughout the heart cycle to spot the best actuation timing, (**B**) fine-tuning where the start (S) and end (E) of the activation are fine-tuned to optimize the assistance to the heart. PLV: pressure in the left ventricle.

First, we defined a reference time for the start of activation. This time was set to have the decompression pressure wave, created by the activation of the DEA, arriving at the aortic valve at opening. Similarly, the end of activation is chosen to be in synchronization with the aortic valve closure. This leads to an earlier activation compared to the opening of the valve because of the propagation time of the pressure wave from the location in the graft to the aortic valve. The synchronization between the pacemaker, the opening of the valve and the actuation of the DEA is described in more depth in Martinez *et al*. [11]. We then defined 2 protocols based on this reference time as shown in Fig. 2A and B. In the 1st protocol A, the activation profile of the DEA remained fixed, and we changed the start of activation by phase shifting it through the heart cycle in % of the heart cycle duration. With this protocol, we obtain insight into the influence of the activation of the DEA throughout the heart cycle. In the 2nd protocol B, we focused on fine-tuning the activation of the DEA. The 1st protocol allowed to define the starting time that results in the optimal assistance to the heart. From this starting point, we then slightly change the start and end of activation to find the overall best configurations that maximize the assistance to the heart.

For each DEA, the 2 protocols were performed at a given voltage. We proceeded with incremental voltage steps: if the DEA sustained the experiment, we increased the voltage and carried out the 2 protocols at this higher voltage. We continued this increase until the DEA suffered electrical breakdown.

### Statistical analysis

Cardiac parameters were recorded during baseline (60 heart cycles before and 60 heart cycles after device-actuated period) and during actuation of the device (60 heart cycles). Only 40 consecutive heart cycles, 20 baseline and 20 device-actuated (either with baseline cycles before actuation followed by the 1st cycles of actuation or with the last cycles of actuation followed by the 1st baseline cycles after actuation), for each device protocol were analysed using MATLAB (MathWorks, Natick, USA) to limit the effect of irregular events, such as arrhythmia or nonstable haemodynamic conditions in the analysis. The 20 + 20 heart cycles were consecutive, and for each of the cycles, pressure, flow and volume parameters were calculated. Additionally, the mean values and standard deviations for the baseline (20 cycles) and device-actuated cycles (20 cycles) were calculated for each parameter. To compare actuated cycles with baseline among all animals and all devices, the mean values were normalized to baseline for each observation (20 + 20 cycles) by calculating the change compared to baseline in percentage as 100 × (mean of actuated cycles – mean of baseline cycles)/(mean of baseline cycles). For each device protocol, the normalized observations were grouped in range of actuation timings (phase shifts) and the Wilcoxon signed-rank test (signrank in MATLAB) was performed to compare the device-actuated cycles with the baseline cycles for all DEAs and all animals. The same test was used to compare the different groups of actuation timing. To compare the different animals, we performed the Kruskal–Wallis test (kruskalwallis in MATLAB), and if significant, we performed a multiple comparison (multcompare in MATLAB) to test which animals were significantly different. A *P*-value below 0.05 was considered significant.

## RESULTS

All the pigs were implanted with the pre-stretched DEA. At baseline, the 5 included pigs had aortic pressures in the range of 51.3–118.0 mmHg (mean: 88.3 ± 11.0 mmHg) and 24.6–78.3 mmHg (mean: 46.7 ± 13.8 mmHg) systolic and diastolic pressure, respectively. The cardiac output ranged from 1.2–5.7 l/min (mean: 3.1 ± 0.9 l/min). Supplementary Material, Table S1 shows an overview of the used DEAs, the actuation voltages and the performed protocols for each animal. All recordings performed in animal 2 were excluded from the analysis due to poor data quality (e.g. signal noise), arrhythmic events or nonstable haemodynamic condition of the animal. Additionally, the PV catheter signals were difficult to measure (due to e.g. motion artefacts and pressure sensor touching the ventricular wall) leading to only a few recordings in animal 5 with an acceptable signal quality. No device-related adverse events were observed in any animal, and no animal died during surgery. Across the animals, a significant difference of actuation response was observed (*P* < 0.05). However, across the animals with comparable actuation voltage (animals 3 and 4), no statistical significance was observed (*P* > 0.05). The different response of animals 1 and 5 compared to animals 3 and 4 can be attributed to the lower actuation voltage (animal 1) and to the loss of performance of the DEAs due to device extensive usage (animal 5) leading to lower responses to actuation. The statistical analysis across the animals can be seen in the Supplementary Material Fig. S1.

### Improved assistance to the heart: up to 16%

**Figure 3:**
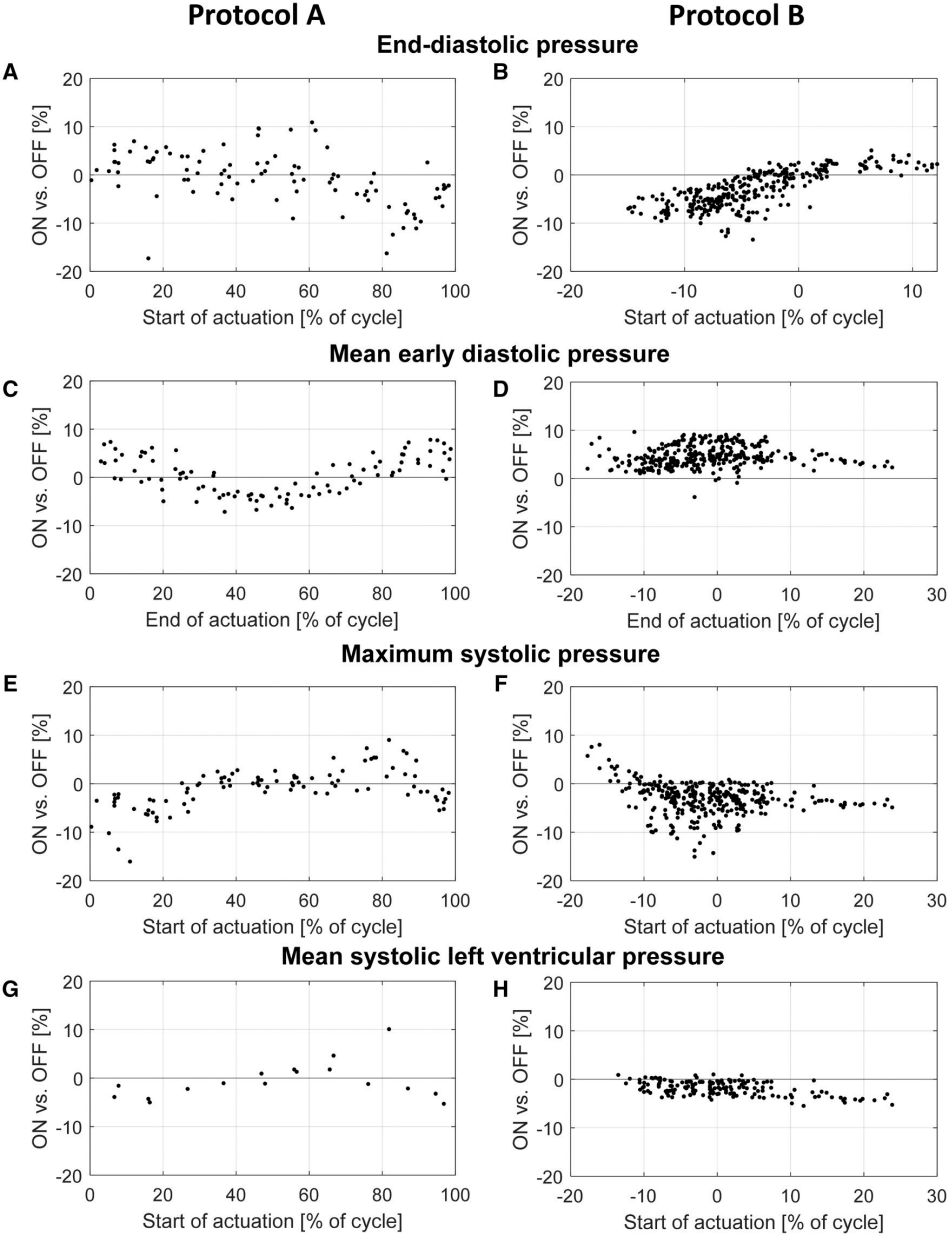
Overview of the impact of DEA actuation timing (ON: device on) on aortic (**A**–**F**) and left ventricular (**G** and **H**) pressure parameters compared to baseline (OFF: no actuation) calculated as [100 × (device-actuated – baseline)/baseline]. (**A**, **C**, **E**, **G**) shows the impact of phase shifting (protocol A) and (**B**, **D**, **F**, **H**), the impact of fine-tuning (protocol B) of the actuation timing. DEA: dielectric elastomer actuator.

The best DEA-assistance results are achieved with counterpulsation (start of actuation around opening of the aortic valve and end of activation around the closure of the aortic valve). Figure 3 show all results from the 2 protocols (A: phase shifts, B: fine-tuning) and all animals, and how the different parameters are affected by the DEA actuation timing. Table 1 shows the mean values and the standard deviations of the same parameters. The *P*-values for significant differences compared to baseline within each group and the number of recordings are presented in Supplementary Material, Table S2. The results from protocol A (Fig. 3A, C, E and G) show that the optimal start of activation lies between 90% and 10% delay, thus defining the study area for protocol B (fine-tuning). Compared to baseline, the end-diastolic pressure (Fig. 3A and B) decreases by up to 13 ± 4.0% [start of actuation before aortic valve opening: –30% to 0% of heart cycle (*P* < 0.05), range: 2–10% decrease, Table 1], the mean early aortic diastolic pressure (Fig. 3C and D) increases by up to 10 ± 3.5% [end of actuation around aortic valve closure: –20% to 10% of heart cycle (*P* < 0.05), range: 3–5% increase, Table 1], the maximum systolic pressure (Fig. 3E and F) decreases by up to 16 ± 3.6% [start of actuation around aortic valve opening: –10% to 30% of heart cycle (*P* < 0.05), range: 2–7% decrease, Table 1] and the mean systolic left ventricular pressure (Fig. 3 G and H) decreases by up to 5 ± 3.8% [start of actuation around aortic valve opening: –10% to 10% of heart cycle (*P* < 0.05), range: 1–3% decrease, Table 1]. The different groups of DEA actuation timing were found to be significantly different (*P* < 0.05) for both protocols (see Supplementary Material Fig. S2 for more detail).

**Table 1:** Overview of the mean values and standard deviations of measured differences between baseline and device-actuated heart cycles [100 × (device-actuated – baseline)/baseline] in the haemodynamic parameters for protocol A (phase shifting) and protocol B (fine-tuning)

Actuation (ON or OFF) (%)	Protocol A: phase shift									
	0–10	10–20	20–30	30–40	40–50	50–60	60–70	70–80	80–90	90–100
End-diastolic pressure (ON)	1.82 ± 2.6*	1.28 ± 7.2*	1.65 ± 3.0*	0.38 ± 3.8*	4.02 ± 4.4	–0.31 ± 5.1*	1.27 ± 6.3*	–3.04 ± 1.8	–9.60 ± 3.1	–3.57 ± 3.2
Mean early diastolic pressure (OFF)	3.77 ± 2.8	2.13 ± 3.1*	–0.26 ± 3.2*	–2.85 ± 2.5	–4.45 ± 1.2	–3.91 ± 1.5	–1.43 ± 2.3*	1.17 ± 2.2*	3.54 ± 2.2	4.61 ± 2.8
Maximum systolic pressure (ON)	–5.48 ± 4.0	–6.68 ± 3.6	–2.66 ± 2.5	0.95 ± 1.1	0.34 ± 1.3*	0.56 ± 1.2*	0.81 ± 2.3*	3.65 ± 3.4*	3.23 ± 3.5	–3.02 ± 1.5

	Protocol B: fine-tuning				
Actuation (ON or OFF) [%]	–15 to -10	–10 to -5	–5 to 0	0 to 5	5 to 10
End-diastolic pressure (ON)	–6.60 ± 1.7	–5.25 ± 2.4	–2.05 ± 3.1	0.82 ± 1.9	2.32 ± 1.2
Mean early diastolic pressure (OFF)	3.64 ± 2.1	4.16 ± 1.8	4.94 ± 2.5	5.31 ± 2.2	5.19 ± 1.8
Maximum systolic pressure (ON)	–2.51 ± 2.2	–2.23 ± 2.7	–3.24 ± 3.1	–3.21 ± 3.6	–6.36 ± 3.9
Mean systolic LV pressure (ON)	–0.81 ± 1.1*	–1.22 ± 1.2	–2.42 ± 1.2	–2.48 ± 1.3	–3.52 ± 1.5

*Non-significant change

For each protocol, the results have been grouped in range of actuation timings (phase shifts) to showcase the optimal timing for the start (ON) and end (OFF) of DEA actuation. Depending on which type of parameter, the actuation timing considered is either ON or OFF. For protocol A, the mean systolic left ventricular (LV) pressure was not included due to too few recordings with working LV pressure measurement to perform statistics.

### Overall best results for the aortic pressure parameters

**Figure 4:**
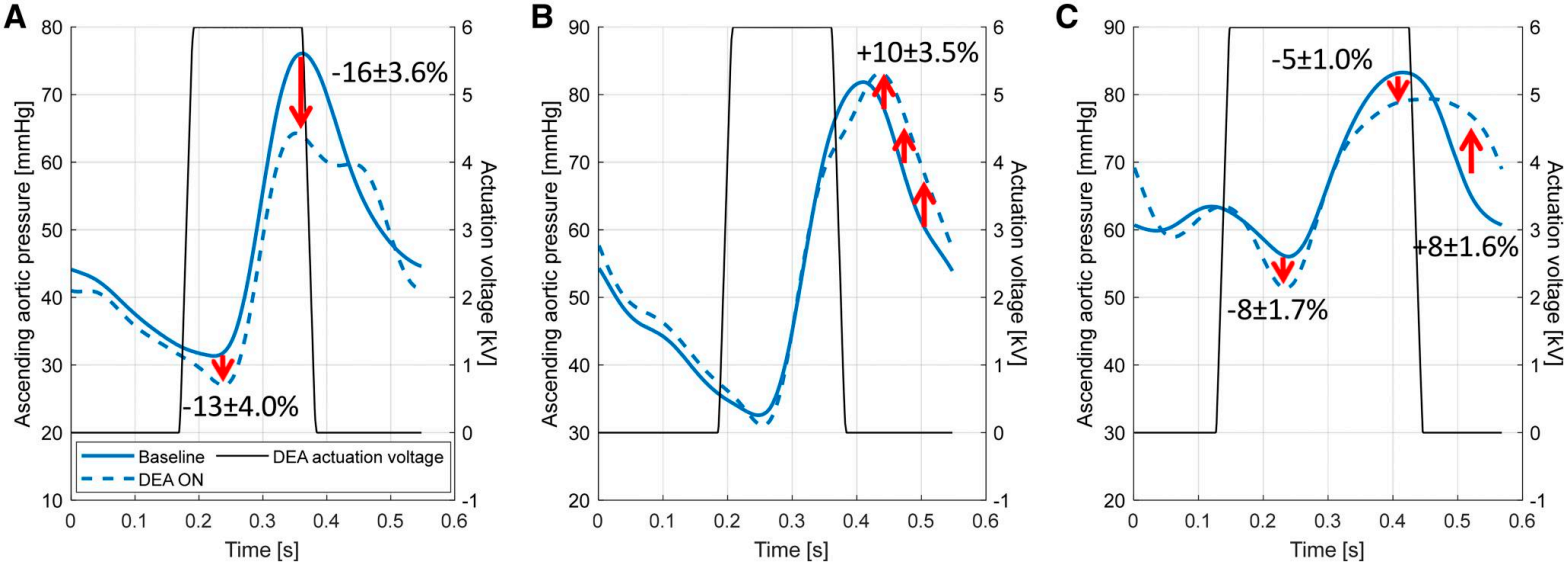
Best results of (**A**) end-diastolic pressure (–13 ± 4.0%) and maximum aortic systolic pressure (–16 ± 3.6%) decrease and (**B**) mean early aortic diastolic pressure (10 ± 3.5%) increase. (**C**) Overall best results for all 3 parameters simultaneously with start of actuation at –8% of heart cycle before aortic valve opening and end of actuation at 0% of heart cycle before aortic valve closure. DEA: dielectric elastomer actuator.

Figure 4 shows examples of the overall best results observed during all experiments (interindividual) performed in this study for the ascending aortic pressure parameters. Figure 4A shows the case (animal 3, DEA 4, 6 kV, protocol B, start and end actuation timing: –4%, –3%) with the relative largest reductions in maximum systolic aortic pressure and end-diastolic pressure, and Fig. 4B shows the relative largest increase in mean early aortic diastolic pressure (animal 3, DEA 4, 6 kV, protocol B, start and end actuation timing: –5%, –11%). Figure 4C shows the relative best overall assistance of the same 3 parameters observed simultaneously (animal 4, DEA 5, 6 kV, protocol B, start and end actuation timing: –8%, 0%).

### Influence of actuation timing on the pressure–volume characteristics of the left ventricle

**Figure 5:**
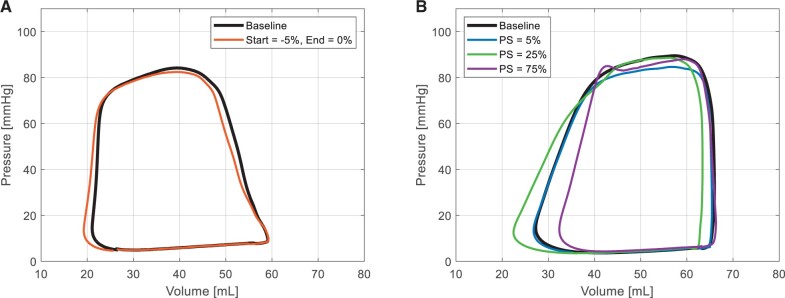
Examples of pressure–volume curves of the left ventricle during DEA support at (**A**) counterpulsation compared to baseline and at (**B**) different actuation timings compared to baseline. Baseline: device turned off. PS: phase shift.

The PV characteristics of the left ventricle is influenced by the DEA actuation timing. Figure 5 shows the PV characteristics during counterpulsation (Fig. 5A) and for 3 different phase shifts (actuation timings) (Fig. 5B) observed in animal 5 (note: due to limited recordings with working PV measurements these results are single observations). During counterpulsation, the PV loops shifts to the left (Fig. 5A), the mean systolic left ventricular pressure decreases (2%) and the stroke volume increases (4%). For the phase shifts (Fig. 5B), an actuation at the beginning of systole (phase shift 5%) lowers the mean systolic left ventricular pressure (5%) without altering the stroke volume and thereby reduces the stroke work of the ventricle (4%). An actuation during end of systole (phase shift 25%) sucks more blood out of the ventricle just before aortic valve closure and increases the stroke volume (6%) with a small decrease in mean systolic left ventricular pressure (3%), leading to a larger stroke work. A deactivation during end of systole (phase shift 75%), however, pushes blood towards the ventricle, while the aortic valve is still open. This reduces the stroke volume significantly (15%) without altering the mean systolic left ventricular pressure leading to a significant decrease in stroke work (14%). In this case, the device hinders the work of the heart.

### Pre-stretching the dielectric elastomer actuator increases the energy provided to the cardiovascular system

**Figure 6:**
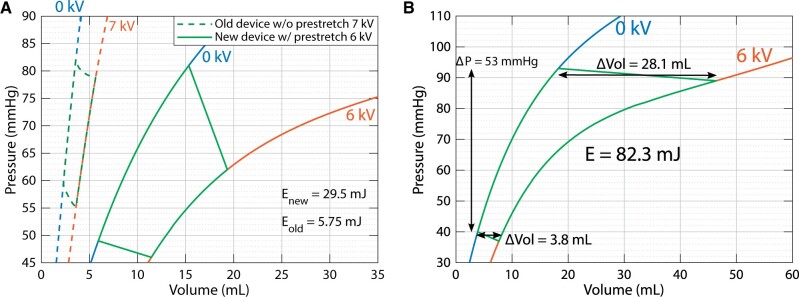
(**A**) Energy provided by the DEA *in vivo* and comparison between the new design using pre-stretch (E_new_) and the older one without pre-stretch (E_old_). With almost similar pressure conditions, the new design provides more than 5 times the energy of the older design. (**B**) Representation of 1 particular *in vivo* case that showcases the maximum energy provided by the DEA. The maximum energy is then of 82.3 mJ with differences of volume (ΔVol) during activation and deactivation of 3.8 ml and 28.1 ml, respectively.

The energy provided by the DEA during the *in vivo* experiments were estimated from the *in vivo* pressure measurements and through comparison with the *in vitro* tests performed at static pressure levels. In Fig. 6A, we compare the estimated energy of the pre-stretched DEA to the previous DEA design without pre-stretch [11] at similar pressure levels *in vivo.* The here reported pre-stretched DEA supplies 29.5 mJ against 5.75 mJ for the previous DEA design without pre-stretch with a voltage lower than before, 6 kV against 7 kV. In Fig. 6B, we represent the recording with the (interindividual) largest estimated energy provided during the current *in vivo* experiments (animal 3, DEA 4, protocol A, 6 kV, start and end actuation timing: 2.3%, –1.8%). In this case, the difference of pressure inside the DEA between activation and deactivation is almost 50 mmHg leading to energy as high as 82.3 mJ. Moreover, at 90 mmHg, the volume of blood displaced during deactivation is very high climbing up to 28 ml, almost 10 times more than with the previous design.

## DISCUSSION

We presented a new design for the DEA and a new implantation technique was introduced with a graft bypass between the ascending aorta and the descending aorta and with the DEA implanted in it. This new setup was tested *in vivo* in a porcine model (*n* = 4), while monitoring haemodynamic parameters such as the aortic and left-ventricle pressures, blood flow and volume of the left ventricle.

### Better assistance to the heart with pre-stretched dielectric elastomer actuator

The new pre-stretched DEA design improved the energy provided to the heart and the volume of blood displaced during activation and deactivation of the device. Additionally, the voltage required to activate the DEA was reduced. In this *in vivo* work, we show improvement of the assistance to the heart compared to our previous DEA design without pre-stretch [11]. For the best cases shown here, the end-diastolic pressure and peak systolic pressure in the aorta were reduced 2.5 and 6.5 times more compared to the reduction observed with our previous design, and the early diastolic aortic pressure increased 5 times more compared to the previous design. Moreover, during this experiment, we could exploit some of the data from the PV catheter inserted in the left ventricle to showcase the influence of the DEA on the PV characteristics. We found out that for the same activation timing that led to the optimized results presented before, the work of the left ventricle was reduced due to the lowering of the pressure (afterload). Additionally, a small increase in stroke volume was observed. This is comparable to the effects reported during IABP support. [17–19]. However, by choosing a different start for the activation, different effects can be achieved: increase or reduction in left ventricular pressure, stroke volume and stroke work. We must add that although these effects are very significant on the displayed results, they lack statistical evidence as the measurement was not exploitable for many of the tested DEAs due to bad positioning of the PV catheter. Nonetheless, the actuation timing could still be tuned to fit the desired effect for the patient.

This constitutes a notable difference compared to other counterpulsation systems especially IABPs. The DEA is never obstructing the blood flow and thus can be actuated in different parts of the heart cycle. On the contrary, activation is limited to only diastole for IABPs. Furthermore, in terms of assistance, the new setup presented here allows to reach the levels provided by IABPs regarding the decrease in end-diastolic pressure. Kolyva *et al*. [20] reported reduction in end-diastolic pressure up to 13.7% and similar results were presented in Lu *et al*. [21] with 13.9% reduction for IABP. The authors also reported an increase of the peak diastolic aortic pressure of 26.7%. For para-aortic balloon pumps, decrease in end-diastolic pressure of 34.7% and increase of 39.2% of the peak diastolic aortic pressure are demonstrated [21]. However, for this latter device, the inflation can deflect the flow of blood from its natural path as the balloon is implanted outside of the aorta.

The typical values of volume inflation in these systems range from 30 to 50 cm^3^ for IABPs and 40 cm^3^ for para-aortic balloon in Lu *et al*. [21] allowing more assistance to the heart than with our device. We showed that the maximum displacement of volume, for our device, was 28 cm^3^, but the typical range goes from 10 to 25 cm^3^. These results depend greatly on the haemodynamic parameters and, more importantly, on the pressure range at which the DEA is working. For clinical application where the pressure range might be higher, our actuator could provide more volume displacement and surpass typical IABP assistance results.

### Strengths and weaknesses of the new surgery configuration

In the new surgical configuration, all blood ejected from the heart passes through the graft and the DEA. In addition, the DEA is located closer to the aortic valve. Both these changes should improve the assistance to the cardiovascular system: (i) they ensure a better synchronization of the device with the cardiac cycle due to its proximity to the aortic valve, (ii) the counterpulsation efficiency and blood volume displacement are maximized because the flow in the actuator is increased and the pulse propagation in other arteries is minimized and (iii) we can expect the blood flow in the coronary arteries to be maximized [22, 23]. However, adding a rigid graft bypass in the blood flow increases the afterload (resistance) of the left ventricle and potentially the workload compared to the native configuration. Furthermore, the presented surgery remains very invasive and complex, although it allows more easy replacement of defective devices (by reopening the clamp in the ascending aorta and clamp the graft during exchange). This experimental surgery, however, remains a testing setup that allows to emulate conditions closer to the final application, i.e. implantation in the human ascending aorta, but does not represent the final clinical application.

### Perspectives

The presented results show a significant improvement compared to previous designs and showcase the interest of this solution for future clinical application. The proposed DEA-based cardiac assist device only require electric stimulation and research is currently ongoing to develop transcutaneous wireless power transfer in order to have a fully implanted device (without drivelines passing through the skin) [24]. Furthermore, some aspects of the systems are being refined to be more adapted to clinical applications. We are currently working on reducing the invasiveness of the surgery by wrapping the DEA around the aorta (extra-aortic) and on synchronization of the actuation with electrocardiogram electrodes. The latter especially could be beneficial in chronic experiments.

## CONCLUSION

In this work, we propose a new design of a DEA-based cardiac assist device. The new pre-stretched DEA was able to supply more energy and displace more volume of blood than the previous design without pre-stretch. The new design combined with the surgical implantation of the device helped improving the assistance to the heart. Furthermore, we demonstrated a new operating mode to assist the heart with the same device as when changing the activation timing, the activation of the device can increase the stroke volume or reduce the stroke work of the left ventricle.

## Supplementary Material

ivae027_Supplementary_Data

## Data Availability

All data are available upon request to the corresponding author.

## Notes

Presented at the EACTS 2023 annual meeting, Vienna, 6^th^ October 2023.

## ABBREVIATIONS

ACP: Aortic counterpulsation
DEA: Dielectric elastomer actuator
HF: Heart failure
PV: Pressure–volume
VAD: Ventricular assist device
