# Supplementary material for: Novel para-aortic cardiac assistance using a pre-stretched dielectric elastomer actuator
Source: Interdiscip Cardiovasc Thorac Surg. 2024 Feb 28;38(3):ivae027. doi: 10.1093/icvts/ivae027 (PMC10955250; doi:10.1093/icvts/ivae027)
Supplement: ivae027_Supplementary_Data [file ivae027_supplementary_data.docx]

Supplementary Materials for

Novel para-aortic cardiac assistance using a pre-stretched dielectric elastomer actuator

**Authors:** Silje Ekroll Jahren^1,2*^, Thomas Martinez^1*^, Armando Walter^1^, Francesco Clavica^1,2^, Paul Philipp Heinisch^3,4^, Eric Buffle^5^, Markus M Luedi^6^, Jurgen Hörer^4^, Dominik Obrist^2^, Thierry Carrel^7^, Yoan Civet^1^, Yves Perriard^1^

* These two authors contributed equally to this work

**Anaesthesia**

Upon reaching the testing facility, the animals underwent a pre-anaesthetic clinical examination. Initial assessments included baseline measurements of heart rate, respiratory rate, and temperature. Subsequently, the animals were sedated using intramuscular ketamine (10 mg/kg), dexmedetomidine (15 mcg/kg), and morphine (0.2 mg/kg). With oxygen supplementation via a facial mask, an intravenous cannula was placed in the marginal auricular vein. Following the preparation of the surgical site, general anaesthesia was induced using ketamine (1 mg/kg) and propofol to effect (1-6 mg/kg). Amoxicillin and clavulanic acid (20 mg/kg) were administered after the induction of general anaesthesia. After tracheal intubation, anaesthesia was maintained with sevoflurane in a mixture of oxygen and compressed air. End-tidal sevoflurane levels were adjusted to ensure an adequate depth of anaesthesia up to the minimum alveolar concentration (MAC) of 2.7%. Prior to thoracotomy, additional analgesia was provided through the injection of ropivacaine 0.75% (up to 2 mg/kg) and morphine 0.1 mg/kg via a spinal catheter inserted through the lumbosacral space. Positive pressure ventilation commenced post-tracheal intubation, with a positive end-expiratory pressure (PEEP) of 5 cmH2O and a tidal volume of 8-12 mL/kg body weight, targeting a PaCO2 of 40-45 mmHg. Central venous pressure was recorded by inserting a jugular four-lumen catheter, and rapid volume infusion was facilitated through an 8.5 French catheter in the external jugular vein. Catheterization of the carotid and saphenous arteries allowed continuous monitoring of arterial blood pressure. Throughout general anaesthesia, various parameters, including heart rate, respiratory rate, arterial oxygen saturation, capnography, invasive blood pressure, oesophageal temperature, inspired and expired fractions of gases (air, etCO2), central venous pressure, and EEG, were monitored using a multi-modular monitor (S/5 Critical Care Monitor®; Datex-Ohmeda, GE Healthcare, Helsinki, Finland). A mean arterial blood pressure (MAP) of 65 mmHg was maintained during anaesthesia, with hypotension addressed through the use of colloids and titration of inotropes/vasopressors. To prevent arrhythmias induced by heart manipulations during thoracotomy, amiodarone (3-5 mg/kg) was administered. Crystalloids and/or colloids were administered throughout the anaesthesia duration. At the conclusion of the experiments, Pentobarbital (100 mg/kg) was intravenously injected while the animals were under general anaesthesia. Confirmation of death was based on EEG and ECG silence.

**Table S1:** Overview of all the measurements performed and all the devices and protocols tested for each animal implanted with DEAs during the i*n vivo* experiments. Protocol A: phase shifting of actuation. Protocol B: fine-tuning of actuation timing.

|  | **Overview of animals, protocols and devices** | | |
| --- | --- | --- | --- |
| **Animal [-]** | **DEA [-]** | **Voltage [kV]** | **Protocol [-]** |
| **1** | 1 | 4,75 | A |
|  |  |  | B |
|  | 2 | 4,75 | A |
| **2** | Not used in analysis due to data quality | | |
| **3** | 3 | 5,00 | A |
|  |  |  | B |
|  |  | 5,50 | A |
|  | 4 | 5,00 | B |
|  |  | 6,00 | A |
|  |  |  | B |
|  |  | 6,50 | B |
| **4** | 5 | 5,00 | A |
|  |  |  | B |
|  |  | 5,50 | A |
|  |  |  | B |
|  |  | 6,00 | A |
|  |  |  | B |
|  | 6 | 5,50 | A |
|  |  |  | B |
|  |  | 6,00 | A |
|  |  |  | B |
| **5** | 7 | 5,50 | A |
|  |  |  | B |
|  | 8 | 4,50 | B |
|  | 9 | 4,50 | B |
|  |  | 5,00 |  |
|  |  | 5,50 |  |
|  | 10 | 5,00 | B |

**Table S2:** Overview of the p-values indicating the significance of the changes in hemodynamic parameters (Wilcoxon signed-rank test) and number of recordings used to perform the statistics for Table 1.

|  | **Protocol A: phase shift** | | | | | | | | | | | | | | | | | | | | | | |
| --- | --- | --- | --- | --- | --- | --- | --- | --- | --- | --- | --- | --- | --- | --- | --- | --- | --- | --- | --- | --- | --- | --- | --- |
| **Actuation (ON or OFF) [%]** | 0-10 | | 10-20 | | 20-30 | | | 30-40 | | | 40-50 | | | 50-60 | 60-70 | | | 70-80 | | 80-90 | | 90-100 | |
| **End-diastolic pressure (ON)** | **0.064 (10)** | | **0.232 (10)** | | **0.105 (10)** | | | **0.820 (9)** | | | **0.039 (8)** | | | **1.000 (10)** | **0.910 (9)** | | | **0.031 (7)** | | **0.002 (10)** | | **0.010 (10)** | |
| **Average early diastolic pressure (OFF)** | **0.020 (9)** | | **0.105 (10)** | | **1.000 (10)** | | | **0.020 (9)** | | | **0.004 (9)** | | | **0.004 ( 9)** | **0.156 (7)** | | | **0.123 (11)** | | **0.002 (10)** | | **0.004 (10)** | |
| **Maximum systolic pressure (ON)** | **0.002 (10)** | | **0.002 (10)** | | **0.010 (10)** | | | **0.039 (9)** | | | **0.426 (9)** | | | **0.193 (10)** | **0.426 (9)** | | | **0.078 (7)** | | **0.027 (10)** | | **0.002 (10)** | |
|  |  |  | | | |  | | |  | | |  | | | |  |  | |  | |  | |  |
|  | **Protocol B: fine-tuning** | | | | | | | | | | | | | | |  |  |  |  |  |  |  |  |
| **Actuation (ON or OFF) [%]** | -15-10 | | | -10-5 | | | -5-0 | | | 0-5 | | | 5-10 | | |  |  |  |  |  |  |  |  |
| **End-diastolic pressure (ON)** | **<0.001 (33)** | | | **<0.001 (122)** | | | **<0.001 (97)** | | | **0.002 (33)** | | | **<0.001 (24)** | | |  |  |  |  |  |  |  |  |
| **Average early diastolic pressure (OFF)** | **<0.001 (28)** | | | **<0.001 (76)** | | | **<0.001 (83)** | | | **<0.001 (78)** | | | **<0.001 (28)** | | |  |  |  |  |  |  |  |  |
| **Maximum systolic pressure (ON)** | **<0.001 (33)** | | | **<0.001 (122)** | | | **<0.001 (97)** | | | **<0.001 (33)** | | | **<0.001 (24)** | | |  |  |  |  |  |  |  |  |
| **Average systolic LV pressure (ON)** | **0.1484 (8)** | | | **<0.001 (69)** | | | **<0.001 (48)** | | | **<0.001 (21)** | | | **0.0039 (9)** | | |  |  |  |  |  |  |  |  |
| *LV: left ventricular* |  |  | | | |  | | |  | | |  | | | |  |  |  |  |  |  |  |  |
| *p-values <0.05 / p-values > 0.05 (number of recordings)* | | | | | | | | |  | | |  | | | |  |  |  |  |  |  |  |  |


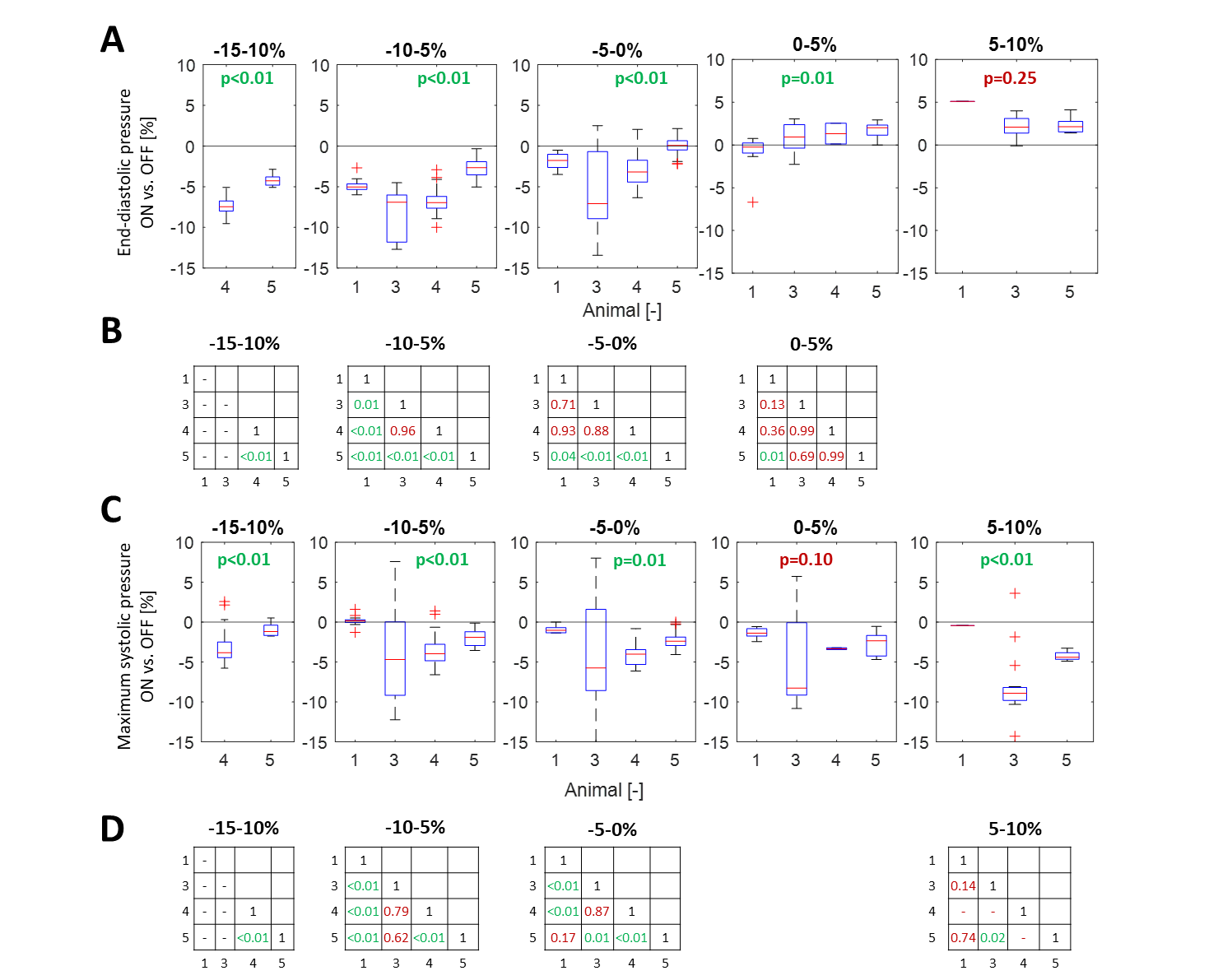


**Figure S1:** Statistical significance across animals for end-diastolic pressure and maximum systolic pressure. A and C shows the data distribution among the four animals represented as boxplots and the p-values of the Kruskal-Wallis test across the animals for each group of DEA actuation timing (same groups as in **Table 1**) for end-diastolic pressure and maximum systolic pressure, respectively. B and D show the p-values of the significance between the different animals for the groups which were significantly different (p<0.05) in A and B, respectively, using multiple comparison (multcompare in MATLAB).


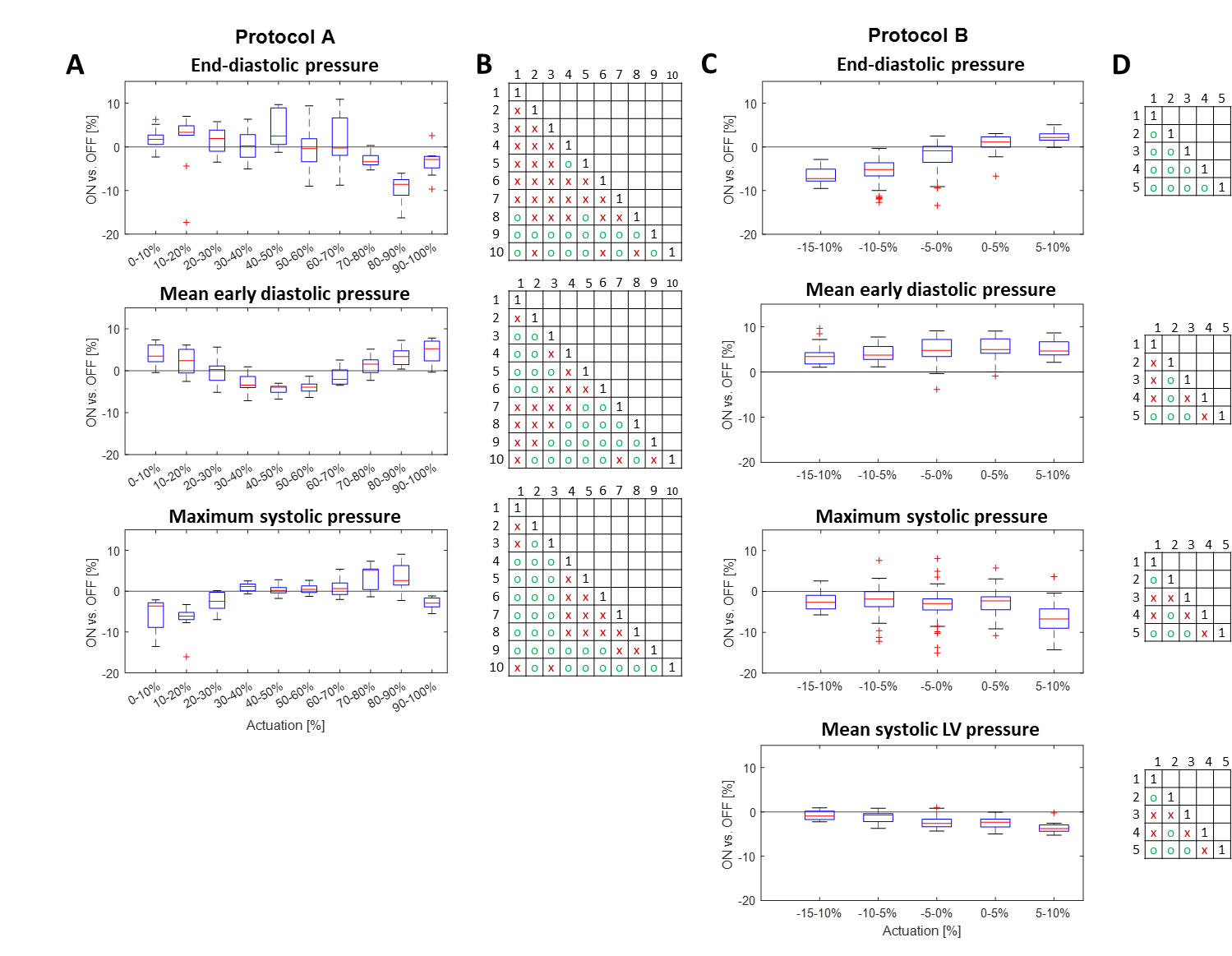


**Figure S2**: Statistical significance across groups of DEA actuation timing (same groups as in **Table 1**) for end-diastolic pressure, mean early diastolic pressure, maximum systolic pressure (protocol A and B) and mean systolic left ventricular (LV) pressure (protocol B). A and C shows the data distribution among the groups represented as boxplots. B and D show the significance between the different groups using Wilcoxon signed-rank test (signrank in MATLAB). Green circles (o) indicate that the groups were significantly different (p<0.05), and a red cross (x) indicates that the groups were not significantly different (p>0.05). The numbers 1-10 and 1-5 represents the groups 0-10% to 90-100% (protocol A) and -15-10% to 5-10% (protocol B), respectively.
